# Contour analysis for interpretable leaf shape category discovery

**DOI:** 10.1186/s13007-019-0497-6

**Published:** 2019-10-07

**Authors:** Jorge Victorino, Francisco Gómez

**Affiliations:** 1 Departament of System Engineering, Universidad Central, Bogotá, 110311 Colombia; 2 Department of System Engineering, Universidad Nacional, Bogotá, 111311 Colombia; 3 Department of Mathematics, Universidad Nacional, Bogotá, 111311 Colombia

**Keywords:** Category discovery, Contour analysis, Interpretability, Leaf shape, Morphological description

## Abstract

**Background:**

The categorical description of leaf shapes is of paramount importance in ecology, taxonomy and paleobotanical studies. Classification systems proposed by domain experts support these descriptions. Despite the importance of these visual descriptive systems, classifications based on this expert’s knowledge may be ambiguous or limited when representing shapes in unknown scenarios, as expected for biological exploratory domains. This work proposes a novel strategy to automatically discover the shape categories in a set of unlabeled leaves by only using the leaf-shape information. In particular, we overcome the task of discovering shape categories from different plant species for three different biological settings.

**Results:**

The proposed method may successfully infer the unknown underlying shape categories with an F-score greater than 92%.

**Conclusions:**

The approach also provided high levels of visual interpretability, an essential requirement in the description of biological objects. This method may support morphological analysis of biological objects in exploratory domains.

## Background

Visual shape description in plants is a very specialized and time-consuming task [1, 2]. The botanist and ecologist require straightforward approaches to communicate relevant information about the plant morphology. The construction of category systems allows the communication of the underlying phenomena and the standardization of biological studies [1]. Visual categorization is also an essential task for botanic manual construction, in which expert knowledge is commonly registered as visual categories [3–6]. In these systems, botanists define key terms accompanied by a visual description of the observed characteristics, with which categories of the shape are established. In systematic biology and taxonomy, experts are extensively trained to perform this task [7, 8].

Leaf categorization based on traditional botanical manuals can be potentially complemented. First, there are exploratory scenarios in which the working hypothesis is related to the analysis of variations of the external morphology on the leaf sheet [1, 9]. These scenarios may require particular categorization systems, not necessarily existing, in the commonly used botanical manuals [9]. Second, human-based labeling may be biased by individual opinions because of the high level of subjectivity implicit in the recognition process of biological objects [10]. Finally, botanical manuals are naturally restricted to narrow biological domains. In principle, these manuals should be constructed for particular cases.

An alternative to characterize plants objectively is digital plant morphology [10]. This approach provides quantitative representations of the object appearance [11–13]. Several plant science problems have been tackled using this method [14], specifically, species classification and characterization of morphological traits in response to changes in environmental or genetic conditions using, for instance, pseudolandmarks or harmonics to characterize the variation of geometric traits of the leaf contours [11–13]. However, despite the utility of these approaches to quantify shape, they are limited to object contours with the same homology [15]. Other tools currently available for performing morphometric measurements, like plantcv [16], morpholeaf [17] or MowJoe [18], do not consider automatic approaches to overcome the construction of visual categories systems to describe shapes in the biological domain.

Besides category discovery, the visual description of forms performed by the expert during biological interpretation may also require complementary information about which were the morphological causes that resulted in the discovered shape classes [19, 20]. This property of geometrical interpretability is fundamental because the knowledge of these causes of the existence of a shape class may potentially help the expert to find explanations of the underlying phenomena, relating the shape of class to adaptation, function, development, among other biological features [9].

To achieve these interpretations, biologists commonly use high-level concepts to characterize leaf shape [21]. For instance, the concept of the type of blade or the kind of margin. Notably, these two concepts are closely related to low and high frequencies of the object contour and are captured by the Fourier transform of the border [13]. This fact suggests the use of the Fourier transform representation for recovering some high-level categories used for the foliar description task. In this work, we propose a novel method to discover the shape categories underlying a set of non-annotated samples based on contour analysis. We show that the use of strategies based on harmonics allows building a representation space that captures some of the high-level features commonly used for botanist and ecologist in the description of geometrical blade information.

## Results

### Capability of the method to recover the original categories

**Fig. 1:**
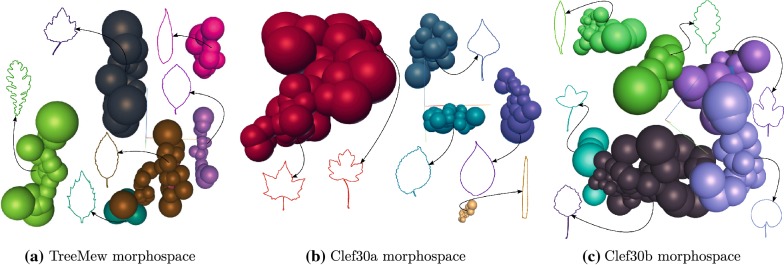
Results of adaptive mean shift clustering for the three evaluated datasets. Each morphospace show spheres and representative leaf prototypes. The center of the spheres represents the position of each leaf sample for the evaluated datasets. The sphere radius is given by the adaptive meanshift algorithm. The spheres that displayed with the same color compose the same leaf shape category. The prototypes were the representative sample of each cluster discovered

Figure 1 shows the morphospace 3D for the evaluated datasets. Each morphospace show spheres and representative leaf prototypes. The center of the spheres represents the position of each leaf sample for the evaluated datasets. The sphere radius is given by the adaptive meanshift algorithm. The spheres that displayed with the same color conformed the same leaf shape category. The prototypes were the representative sample of each cluster discovered. The leaf prototype corresponds to the closest leaf sample using Euclidean distance to the cluster centroid.

Table 1 reports the quantitative performance obtained by using different experimental settings. In particular, two algorithms: meanshift and adaptive meanshift, and two distances: Euclidean and Euclidean plus whitening, which is similar to Mahalanobis distance [22]. This was done in the following combinations: meanshift + whitening, adaptive meanshift + non-whitening, and adaptive meanshift + whitening. As observed, the use of adaptive meanshift and whitening resulted in the highest performance for the three explored datasets. High values of F-scores were obtained for the three datasets. Figure 1 shows that the proposed representation space locates nearby similar shape samples; additionally, the method was able to separate groups of different species samples. Tables 2, 3 and 4 shows the confusion matrix for the evaluated datasets. The Hickey manual was used to set a name for the groups discovered by the method, as shown in Table 5. The name of each group was composed of two parts. The first part relates to the shape of the sheet defined in the Hickey manual [4]. The second part relates to the margin type, also determined by the Hickey manual [4]. The names assigned to each discovered groups are shown as headings in the confusion matrices, see Tables 2, 3, and 4. In the test datasets the emergence of leaf clusters was evident. Finally, Table 2 reports the method recovered most of the ground truth categories associated with the original species. Additional experiments were performed to establish the limitations of the proposed method, Additional file 1 reports the main results.

**Table 1:** Performance comparison between mean shift + whitening (MS + W), adaptive mean shift + non-whitening (MS + NW) and adaptive mean shift + whitening (MS + W)

Dataset	MS + W	AMS + NW	AMS + W
TreeMew	93% ± 2.1	88% ± 3.5	97% ± 1.4
Clef30a	93% ± 1.4	90% ± 2.4	97% ± 1.4
Clef30b	91% ± 2.3	87% ± 3.8	92% ± 2.8

Table reports the mean ± 1 SD for each performance measurement (F-measure)

**Table 2:** Confusion matrix results for TreeMew dataset using adaptive meanshift plus whitening

Specie-group	1. Elliptic-Dentate	2. Elliptic-Crenate	3. Elliptic-Serrate	4. Oblong-Entire	5. Ovate-Crenate	6. Obovate-Dentate	7. Elliptic-Dentate	8. Elliptic-Dentate
*Ilex aquifolium*	14	0	0	0	0	0	5	1
*Fagus sylvatica*	0	20	0	0	0	0	0	0
*Carpinus betulus*	0	0	20	0	0	0	0	0
*Juglans nigra*	0	0	0	20	0	0	0	0
*Populus alba*	0	0	0	0	20	0	0	0
*Quercus frainetto*	0	0	0	0	0	20	0	0

F-measure score 0.95

**Table 3:** Confusion matrix results for Clef30a dataset using adaptive meanshift plus whitening

Specie-group	1. Ovate-Crenate	2. Elliptic-Dentate, Entire	3. Elliptic-Dentate	4. Ovate-Serrate	5. Special-Entire
*Populus nigra*	30	0	0	0	0
*Ulmus minor*	0	30	0	0	0
*Acer campestre*	0	0	30	0	0
*Platanus hispanica*	0	0	0	30	0
*Ruscus acuelatus*	0	30	0	0	0
*Janiperus oxycedrus*	0	0	0	0	30

F-measure score 0.97

**Table 4:** Confusion matrix results for Clef30b dataset using adaptive meanshift plus whitening

Specie-group	1. Ovate-Crenate	2. Obovate-Dentate	3. Elliptic-Crenate, Entire	4. Elliptic-Entire	5. Oblong-Entire	6. Elliptic-Entire	7. Ovate-Crenate
*Ficus acrica*	24	0	0	0	0	0	6
*Quercus petraea*	0	30	0	0	0	0	0
*Populus tremura*	0	0	29	0	0	1	0
*Cercis siliquastrum*	0	0	4	26	0	0	0
*Phillyrea angustifolia*	0	0	0	0	30	0	0
*Acer monspessulanum*	0	0	21	0	0	9	0

F-measure score 0.92

**Table 5:** Morphological description for the species used in each test group [4]

Species	Shape	Margin	Base	Apex
TreeMew selection				
1. *Carpinus betulus*	Elliptic	Dentate	Rounded	Convex
2. *Fagus silvatica*	Elliptic	Crenate	Concave	Convex
3. *Ilex aquifolium*	Elliptic	Serrate	Convex	Acuminate
4. *Juglans nigra*	Oblong	Entire	Decurrent	Acuminate
5. *Populus alba*	Ovate	Crenate	Rounded	Convex
6. *Quercus frainetto*	Obovate	Dentate	Complex	Complex
Clef30a selection				
1. *Populus nigra*	Ovate	Crenate	Convex	Convex
2. *Acer campestre*	Elliptic	Dentate	Convex	Complex
3. *Ulmus minor*	Elliptic	Dentate	Complex	Convex
4. *Ruscus aculeatus*	Elliptic	Entire	Convex	Acuminate
5. *Platanus hispanica*	Ovate	Serrate	Truncate	Convex
6. *Janiperus axycedrus*	Special	Entire	Complex	Straight
Clef30b selection				
1. *Ficus carica*	Ovate	Crenate	Crodate	Convex
2. *Quercus petraea*	Obovate	Dentate	Convex	Convex
3. *Populus tremura*	Elliptic	Crenate	Convex	Convex
4. *Cercis siliquastrum*	Elliptic	Entire	Lobate	Rounded
5. *Phillyrea angustifolia*	Oblong	Entire	Decurrent	Straight
6. *Acer monspessulanum*	Elliptic	Entire	Cordate	Rounded

This description was obtained by using the Hickey manual

### Qualitative evaluation

**Fig. 2:**
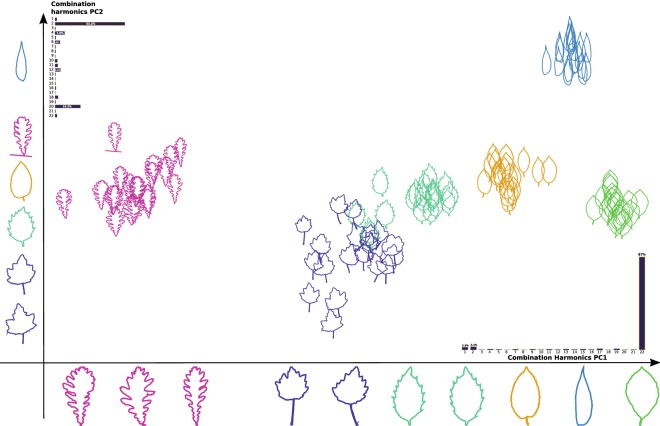
Representation space of leaf shape given by PC1 and PC2 for TreeMew dataset. Each axis represents a principal component and shows its harmonics composite. Different contour leaf samples projected from morphospace are shown under the axis. As observed, x-axis is linked to variations in the margin, while y-axis is linked to blade shape

The proposed method aims also to provide an interpretable representation of the discovered categories. In the experimental setting herein proposed we considered species from six different shape categories from TreeMew dataset [23]. Shapes can be described for the complete leaf or their parts as described in Table 5. These shape categories were proposed using the Hickey manual [4]. This manual contains high level shape concepts related to shape, margin, base and apex. In order to reach high levels of interpretability some leaves were selected from the morphospace to be shown on the representation space axis. For this, we fixed equally spaced points on the axis and the closest sample to these points were shown in the axis, as illustrated in Fig. 2.

**Fig. 3:**
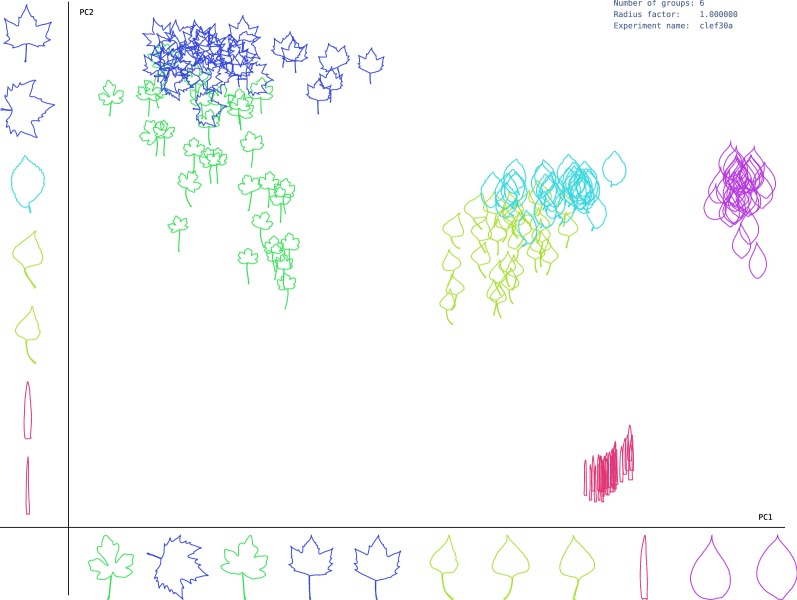
Representation space of leaf shape given by PC1 and PC2 for first Clef selection dataset. Each axis shows different leaf samples projected from the morphospace under its principal component

**Fig. 4:**
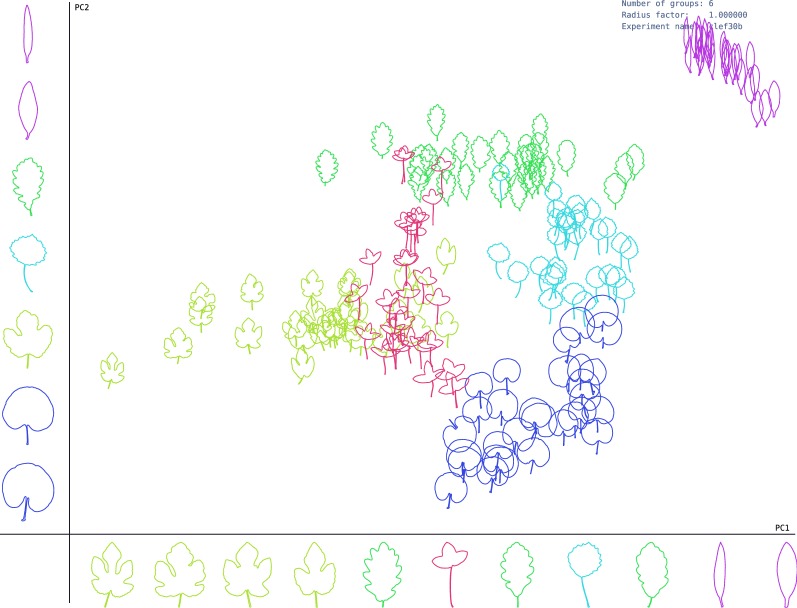
Representation space of leaf shape given by PC1 and PC2 for second Clef selection dataset. Each axis show different leaf samples projected from the morphospace under its principal component

These projections show the morphological variability of the dataset along the main axis. By examining samples in each axis, the shape features that discriminate the groups are identified. As observed, the species with the same margin were closely represented on the first principal component (PC1). Therefore, PC1 represents mainly high-frequency border information that can be linked to these margins. Similarly, the second principal component (PC2) groups species with similar blade shapes, which are projected to the vertical axis from wide to thin form. More specifically, clusters related to species *Carpinus betulus, Fagus silvatica and Ilex aquifolium* are very close in the representation space, as shown in Fig. 2. Interestingly, these species also present high levels of similarity according to the botanical manual, as observed in Table 5. On the other hand, species *Juglans nigra and Quercus frainetto* are far each other, which can also be observed in the proposed representation space. In the ImageClef dataset, Figs. 3 and 4 showed a similar behavior in PC1, corresponding to changes in the margin, while PC2 was related to the leaf width. This result suggests that the method can be used to study margins and shapes simultaneously, resulting in a rich representation.

## Discussion

A new method for the morphological analysis of leaves is introduced. The method allows discovering the categories of leaf shapes in an unlabeled dataset. These categories are interpretable from the biological point of view. The method uses a harmonic representation of the contours, a dimensionality reduction, and an unsupervised clustering strategy. The results show that the strategy identifies categories of leaves related to concepts of margin and foliar lamina. This strategy allows studying sample sets in which the categories are unknown, which may appear in poorly studied biological scenarios.

Results in Table 1 show that the proposed approach may uncover the underlying shape categories for different samples of unlabeled leaves, by using only leaf contour information. In particular, the method provided high values of F1-scores (average $95\%$) in the tasks of discovering previously known shape categories related to the species, by using only unlabeled data. Despite the external morphology variability of the datasets herein explored, which includes different kinds of margin, base and apex, see Fig. 7 and Table 5. The scores and confusion matrices indicate that most of the samples were assigned correctly to the original shape category. Importantly, no prior knowledge about specific shapes resulted in these categories, in contrast to previous approaches that strongly rely on domain expertise, for instance, particular categories of lamina shapes, as commonly found in botanic manuals [3, 4, 6], or individual landmarks located over the leaf border [15]. Importantly, this expert knowledge may not be available for the description of unknown morphological scenarios [17, 24]. Therefore, the proposed approach is relevant for this kind of descriptions. Importantly, this approach provides an objective analysis of the leaf contours without relying on landmarks. Therefore, it can be a valuable tool for interspecific and intraspecific analyzes of the variation of the shape, including in other taxonomic categories (genus, family).

In principle, in unknown biological scenarios, shapes categories are not known beforehand and may differ to ones used for known scenarios [10]. The proposed method can test existing manuals and validate whether they contain the information necessary to classify under-studied leaf morphology or propose a classification form that follows a rigorous mathematical method to avoid ambiguity when choosing a category. To discover these categories, we used a highly flexible low-level representation space that captures biologically meaningful information of the leaf border, in particular, its large and fine variations [25]. The proposed representation captured a broad set of lamina border variations exhaustively in the Fourier harmonics, providing a rich morphospace to represent possibly unknown sample morphologies. We assumed that leaves with similar variations in the border were close in this morphospace. Therefore, shape categories associated with common morphological features are expected to emerge as clusters. Results in Table 2 show that the clusters or shape categories identified in this space, using only the available samples, coincide with the ground truth of shape categories. Remarkably, these categories resemble known shape categories for different classes using only endogenous information of the sample. To our knowledge, this result constitutes the first evidence about the possibility of automatically discovering categories of the shape of biological forms. Alternative approaches have been proposed to discover these categories in natural images [26–28]; however, these approaches have not been explored yet for the discovering of leaf shape categories problem.

**Fig. 5:**
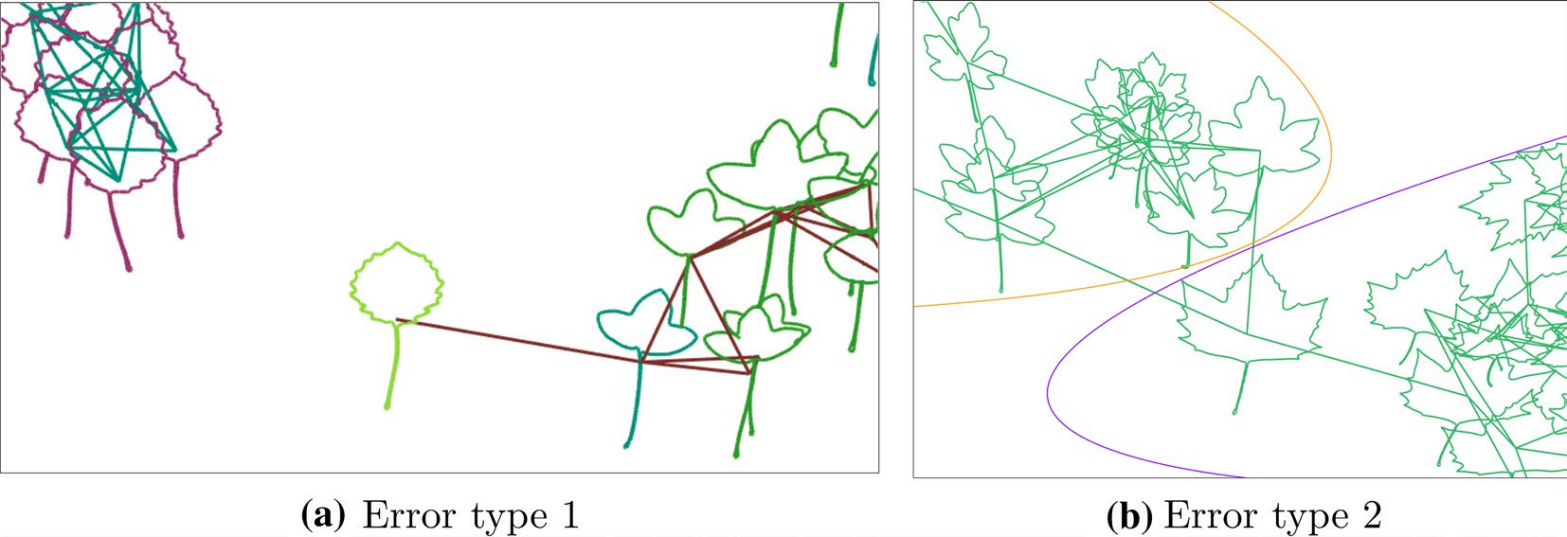
Errors examples, in our approach, leaves with similar shapes form clusters. The lines that appear from the leaf center show how these leaves are connected to shape groups. In the left box (**a**), there are two groups, and in the right box (**b**), there is one. However, on the left, there is a light green leaf that has a shape similar to one group, but it is connected to another. In contrast, in the right box, all the leaves are connected to the same group, but these could be separated into two species by the violet and orange lines

Low performance observed in F1-scores for some of the studied scenarios is linked to two principal causes. First, a high level of morphological overlap among some of the original shape categories. For instance, in the dataset Clef30a the species *Ulmus minor* and *Ruscus acuelatus* have high levels of visual similarity, see Fig. 5b, resulting in a single shape category, see category number two in Table 3. Despite that, the proposed representation was flexible enough to delimitate both categories properly, see curves in Fig. 5b. Importantly, the visualization considered in the model helps to localize and correct errors in the final assignment of the sample category. Second, in some cases, leaf border information was not adequately represented by the Fourier transform, for instance, this representation did not correctly capture border information for samples in specie *Populus tremura* in violet color in Fig. 5a, probably because of the presence of high-frequency information in the serrations [29, 30]. Further investigations may also consider alternative data representations which account for these shape particularities [29–31].

Figures 2, 3 and 4 show that common high level concepts with biological meaning emerged from the representation spaces obtained by PCA projections. Particularly, in the PC1 axis, serrations change from left to right, from serrate margin to entire margin. While in the PC2 axis, leaf shape changes from bottom to top, from wide lamina to narrow lamina. Therefore, we conclude that the major axis relates to the lamina shape concept and minor axis to the border serration. These high-level concepts represent explanations for the shape categories discovered [4–6]. Explainability refers to a human-interpretable description by which the method categorized a shape given a set of unlabeled images [19]. In this case, each discovered category is characterized by a particular combination of lamina and margin shapes. The lamina and serration shape concepts are commonly used by experts to explain leaf shape categories, and they are fundamental for interpreting biological variations [4–6]. These two factors are in the base of leaf descriptive systems of shape categories, and they are commonly used for taxonomical classification, leaf adaptation to environmental conditions, among others.

In order to identify the possible factors associated with the obtained shape categories we performed a posthoc analysis to identify. This kind of analysis is also used in other approaches, for instance, Procrustes and Fourier analysis [15], which consider a subsequent interpretation step aimed to identify sources of variations [15]. In these analyses, experts assign a meaning to observed experimental variations. For instance, correlating shape features with known domain variables. Following a similar approach, we conclude that the shape discovering method provides consistent explanations in biological terms, shape and margin, to the categories discovered. Future work may consider the automatic identification of the concepts that determine the categories and not only rely on the interpreters’ opinion. It is worthy to note that previous approaches to category discovery do not consider the issue of construction of biological explanations to support biological interpretations [27, 28].

In this work, a complex Fourier based representation supported the feature description stage. This transformation provides high levels of visual interpretability [32]. In our experiments, the contours become invariant to geometric transformations, and they were also normalized and centered, as in the Procrustes analysis, but without requiring any landmark. Unlike other approaches of contour analysis, harmonics capture contour variability in the frequency space. Therefore, our approach may serve a tool to analyze this variability in leaves with a different structure. For instance, the approach can be useful when the contours present different lobular compositions, or in sessile leaves, which do not have petiole resulting in open contours. This kind of description is essential also for the description of the external morphology on the leaf sheet [25]. A three dimensional space obtained by PCA embedded the contour representation and a non-supervised clustering algorithm was used on this representation space to infer the corresponding shape categories. The aim here was to reduce the dimensionality of the data in 3D space and provide visualizations and interactivity with the samples in the representation space. As Figs. 2, 3 and 4 show, the leaves were distributed along with the representation space forming dense groups. The distance between a pair of samples was related to how similar samples were and the direction between them, revealed the particular feature that differentiates them. As it happens, when the biologist organizes the obtained sample in leaf categories [13, 33]. This representation allows both a visual representation of the shape information and suitable space to solve the category discovery problem.

The present work has some limitations. First, the proposed method uses only endogenous information of the leaf contour morphology to project the sample into a morphospace, suitable to discover the shape categories. Future work may consider the inclusion of additional information related to the scientific question (for instance, precipitation), which helps to explain the morphological variability of the sample. This complementary information can be included, for example, as an additional part of the feature vector that characterizes each sample. Future work may also consider interaction with the experts to construct a richest morphospace, which enables a post-hoc verification and modification of the proposed categories according to the expert knowledge. Second, increasing the number of categories may difficult the capacity of the method to discriminate the underlying shape groups correctly. As illustrates Additional file 1: Figure S3, when considering a reduced number of PCs, the shape categories cannot be adequately discriminated, and more PCs should be included. Therefore, the inclusion of additional PCs should be considered when the complexity of the database increases.

## Conclusions

In this work, we proposed a novel method to automatically discovery shape categories from the digital image of leaf samples by keeping high levels of visual interpretability of the shape information. The method is based on a complex Fourier representation of the contour, which is embedded in low dimensional representation space. An adaptive clustering method with whitening was used to discover the shape categories. The method was evaluated through the task of predicting the shape categories associated with different plant species. Our results suggest that the proposed method successfully discovers the plant categories by using only leaf shape information providing high levels of visual interpretability.

## Methods

**Fig. 6:**
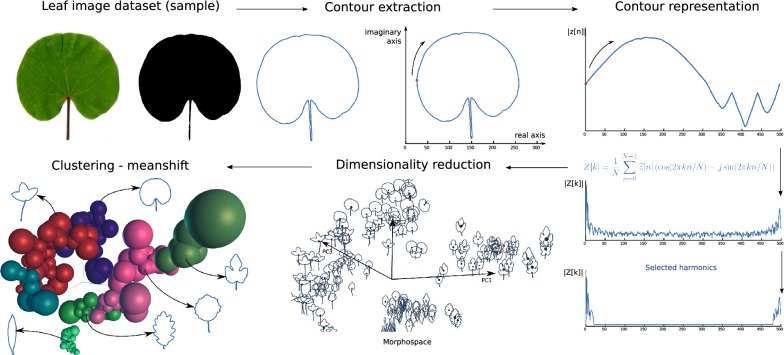
Graphical representation of the strategy for category discovery in leaves dataset. The leaf contours in the dataset were obtained by using binarization and contour extraction. This shape information was represented by a complex Fourier transform. A set of representative harmonics of the leaf information were selected. Following, a dimensionality reduction method was applied to the selected harmonics. Finally, an adaptive kernel density estimation method was used to determine the shape categories

Figure 6 illustrates the proposed method for the construction of interpretable visual categories for a set of images. An image database composed of unlabeled leaves is used as input. The contours of each leaf were extracted by using segmentation and contour extraction algorithms. This information was represented with a complex Fourier transform (CFT), and a set of representative harmonics of the leaf information were selected. Then, a dimensionality reduction method was applied to these harmonics to obtain a three-dimensional morphospace of representation. Finally, an adaptive kernel density estimation method determined the shape categories.

### Contour extraction

The input dataset contained natural images with controlled background. These images were represented in saturation channel because it showed a higher contrast between the leaf lamina and the background. Then, the Otsu method provided a leaf segmentation [34]. A closing morphological operator based in a circular structural element of five pixels of radius removed small holes in the binary image. A tracing algorithm extracted the leaf boundary [35]. This method followed the contour points and returned a two-dimensional vector of vertices. The size of this vector depended on the contour length and the image resolution. In order to have a similar representation among leaves of different sizes, a cubic spline-based interpolation was applied to this array [35]. In particular, $N\,=\,512$ samples uniformly spaced were obtained to represent each contour.

### Contour representation

A *p*-type transformation was used for contour representation, this transform corresponds to a CFT representation of the shape information [36]. Before the CFT, each spatial position of the resampled border (*x*, *y*) was represented as a complex value $z = (x, j y)$, with $j = \sqrt{-1}$. The points in the border conformed a complex discrete signal *z*[*n*], with $n = 1, 2, \ldots , N$. Later, the slopes $\Delta z[n]$ among the adjacent points in *z*[*n*] were computed as $\Delta z[n] = (x[n+1]-x[n], j(y[n+1]-y[n]))$. This representation provides robustness to rotation transformations. The slopes were normalized by the distance $\Vert \Delta z[n]\Vert$ among the neighbor points *n* and $n+1$, as follows: ${\widehat{z}}[n] = \frac{\Delta z[n]}{\Vert \Delta z[n]\Vert }$, with $n = 1, 2, \ldots ,N-1$, this normalization provides invariance to scale transformations. Later, a CFT was applied to the normalized slope signal ${\widehat{z}}[n]$, obtaining:

$$\begin{aligned} Z[k] = \frac{1}{N} \sum _{n=0}^{N-1} {\widehat{z}}[n] e^{-j 2 \pi k n / N}, \end{aligned}$$

where *k* is the harmonic index, $N/2+1$ is the maximum frequency order and *Z*[*k*] is the *k*-th harmonic. For the contour description, it is not essential to use the complete set of harmonics [37]. The first 22 low-frequency harmonics constituted the leaf contour representation. This number of harmonics provided the best compromise between the amount of contour information reconstructed and the size of the representation [32]. This amount of harmonics allowed reconstructions with less than 1% in root mean square difference when considering as reference the original contour [38].

### Dimensionality reduction

Following previous works in the analysis of foliar shapes [10, 32], a dimensionality reduction based on the Principal Component Analysis (PCA) was applied to the selected harmonics. This process was performed by using the Singular Value Decomposition (SVD) of the covariance matrix computed using the complex harmonics [32]. For this, the *q* selected harmonics for the *n* sample contours were organized into a matrix $\Phi \in \mathbb {C}^{q \times n}$. Each entry in covariance matrix $C_{i,k}$ corresponded to the product of the harmonic matrix with its transpose, i.e., $C_{i,k} = \hat{\Phi _i^*} \hat{\Phi _k}^T$, where $\hat{\Phi _i}$ the *i*-th column of *C* and $\hat{\Phi _i^*}$ the hermitian conjugate of $\hat{\Phi _i}$. This covariance matrix was then factorized using SVD, i.e., $C = U \Sigma V^T$, with $\Sigma$ a diagonal matrix containing the singular values, *U* a matrix with orthonormal columns *C* and $V^T$ a matrix with orthonormal rows. The first three columns of *U*, corresponding to the first three eigenvalues, constituted the base for the representation space.

### Clustering

After dimensionality reduction, the category discovery process was performed. For this, the low dimensional data was firstly normalized by applying a whitening transformation in each dimension [39]. A shape category was defined as a cluster emerging in the previously constructed representation space. In this work, two clustering approaches were explored, namely, meanshift [40] and adaptive meanshift [41].

The meanshift algorithm is a non-parametric clustering method for locating the maxima of a density function given *n* discrete data sampled from that function [40]. Given *n* data points $u_{i}$, $i = 1, ..., n$ on a *d*-dimensional space $\mathbb {R}^{d}$, the multivariate kernel density with kernel *K*(*u*) and bandwidth *h* parameter is given by:

$$\begin{aligned} {\hat{f}} = \frac{1}{nh^{d}}\sum _{i=1}^{n}K\left( \frac{u-u_{i}}{h} \right) . \end{aligned}$$

This algorithm provides the modes of the density function, which in our case corresponded to shape categories. The meanshift algorithm directly provides multiple clusters, in contrast to other approaches like k-means which require a definition of the number of classes beforehand. Nevertheless, meanshift results are highly dependent on the bandwidth parameter selection, which indirectly determines the number of classes.

After selecting the first three PCs, the *n* samples were embedded as points in a 3-dimensional morphospace. In this representation space, a set of spheres of radius $\epsilon$ was centered in each point. The sets of overlapping spheres conforming connected components in the representation space were determined and posteriorly associated with shape categories. Note that depending on the sphere radius, a high or low number of shape categories can be found. Therefore, the sphere radius plays a significant role in the shape category discovery procedure.

Two different methods were explored to determine the $\epsilon$ parameter, namely, meanshift [40] and adaptive meanshift [41]. In the meanshift approach a fixed bandwidth *h* is used for all the spheres. In adaptive meanshift the average of the distances to the *k* neighbors for each point is used as a sample dependent sphere radius. More specifically, the Euclidian distances between $u_{i}$ and its first *k* neighbors were averaged, and then used as the sample bandwidth parameter $h_i$ [41]. For this, let $u_i^{(t)}$ the *t*-th nearest neighbor of the point $u_i$ in the morphospace. Then $h_{i,k}$ the average distance the first *k* nearest neighbors was defined as

$$\begin{aligned} h_{i,k} = \frac{1}{k}\sum _{t=1}^k \sqrt{(u_i-u_i^{(t)})^2}. \end{aligned}$$

In the adaptive meanshift algorithm $h_{j,k}$ is used as bandwidth of the point $u_i$.

In the proposed setting, the *k* parameter was obtained experimentally by using as reference the *k* value that recovered the six groups of species that composed TreeMew dataset. This *k* value corresponded to eight and it was used for all experiments.

### Evaluation

#### Leaf image dataset

**Fig. 7:**
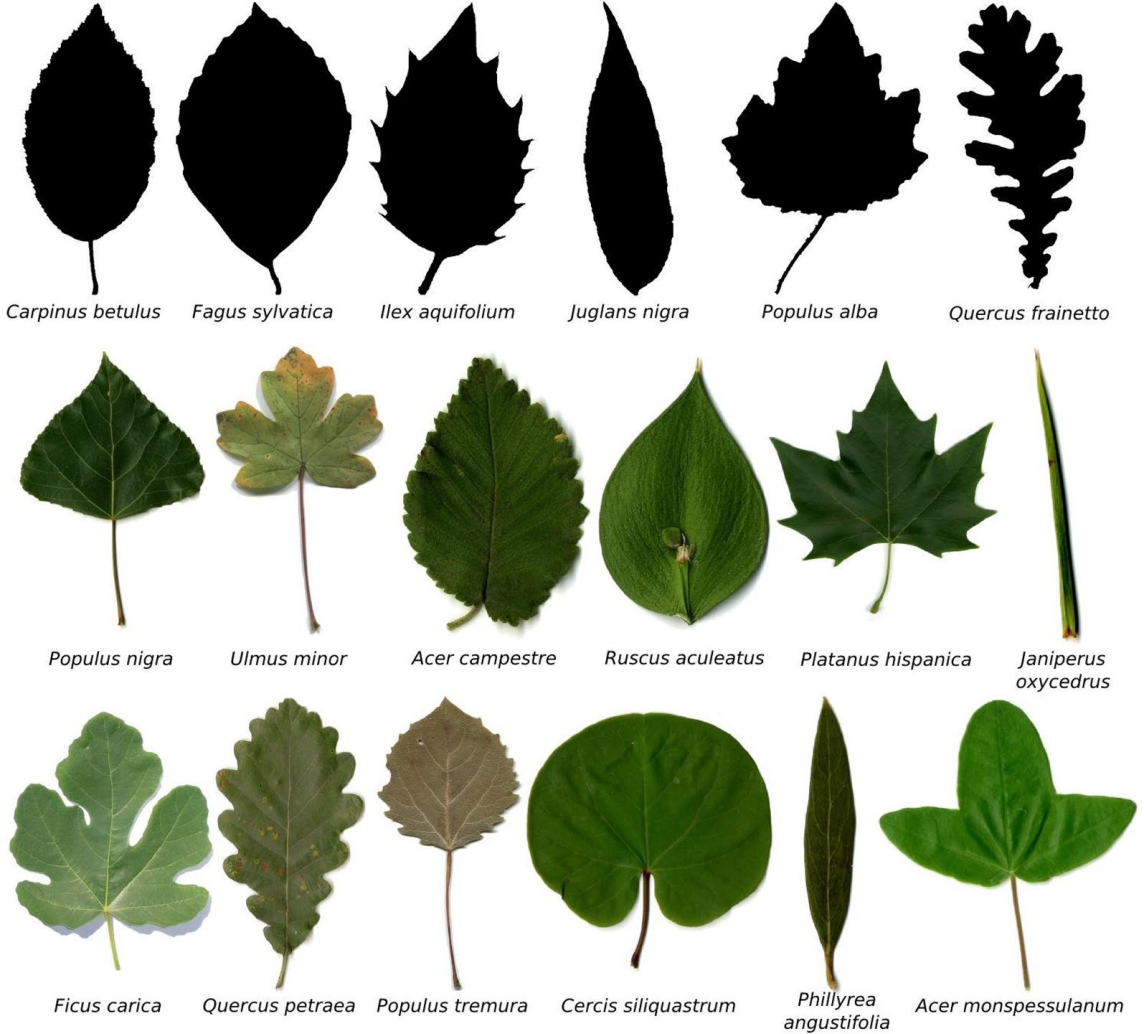
Groups of leaves samples using for testing the method. The image shows the selected species from the TreeMew and ImageClef datasets. The species with the most quantity of samples were selected. The leaves groups were organized in the following way, Top: *Tree leaf database MEW 2010*, middle: *Clef30a* and bottom: *Clef30b*

The category discovery task consists in arranging a non-annotated dataset in a representative set of shape categories and provides for them a coherent explanation in biological terms. There are several public leaf datasets available to study plant species that can be used for evaluation purposes. In this work, two leaf image annotated datasets with information about species with different morphology were selected, namely, TreeMew [23] and ImageClef 2014 datasets [42]. These datasets contain high-quality and quantity isolated leaf image samples, all of them with a controlled background. These conditions helped to extract good-quality contours. Each image in these datasets is annotated with the plant species, which was used as ground truth for the shape category discovery problem. Figure 7 shows a sample of each species selected in this study.

For the quantitative evaluation, the samples were organized in three sets to perform the shape category identification. The TreeMew was used to build a test set (TreeMew) with six groups, with 20 samples per group. Similarly, for the samples in the ImageClef database, two test sets were constructed (Clef30a and Clef30b), each one with six groups, and 30 samples per group. Table 5 shows the corresponding morphological description, which was obtained by using the Hickey manual [5]. As observed, the selected species show differences in their blade shape and margin. It is expected that the proposed method can discriminate samples in different classes using these two criteria. Importantly, these sets have high morphological variability, as Table 5 shows. Therefore, this experimental setting is appropriate for evaluating the category discovery strategy.

#### Experimental settings

The evaluation was twofold: a quantitative evaluation, to assess the method capacity for recovering the original categories, and a qualitative evaluation, to study how the method characterized biologically relevant morphological leaf traits related to the extracted categories.

The shape category discovery problem aims to predict shape categories presented in an unlabeled sample set [27, 28]. We assumed that each plant species corresponds to a different shape category. Under this assumption, the original species of each sample constituted the ground truth for the category discovery problem. A confusion matrix and the corresponding F-score provided quantitative measures of the method performance in the identification of these categories. This last measure considers both the precision and the recall of the class discovery tasks [43]. A leave-one-out scheme was used to study the variability of this performance measurement across different datasets. Once the samples were projected into the reduced representation space, the clustering algorithm was applied for three different configurations of distance and clustering algorithm, namely:

- Data whitening and meanshift algorithm $MS\,+\,W$. Data whitening consists in subtracting the mean and dividing by the deviation of the data in each dimension, similar to the Mahalanobis distance [22].
- Data whitening and adaptive meanshift algorithm $AMS\,+\,W$.
- Data without whitening and adaptive meanshift algorithm.

Finally, a leaf sample per category was projected over the principal components to perform the qualitative assessment. The linear combination of harmonics in each principal component was shown and joined with projected samples for interpretation. The aim here was to recover margin types and blade shape of the leaf samples.

A detailed description of the procedure to reproduce results can be found in Additional file 2 and the source code in Additional file 3.

## Supplementary information

**Additional file 1.** Limits of the category discovery method with multiple samples and species, which reports the results of an experiment aimed to study the limits of the proposed contour analysis method.

**Additional file 2.** Results reproducibility, which shows how to prepare and run the experiments that reproduce the results of the paper.

**Additional file 3.** Source code. This file contains the source code used to produce the paper results. The rar file includes eight Matlab scripts. Before running this code, the instructions given in Additional file 2 must be followed.

## Data Availability

In this work, we have used leaf images datasets of public databases, such as TreeMew [23] and ImageClef [42].
