# Correction to: Contour analysis for interpretable leaf shape category discovery

**DOI:** 10.1186/s13007-019-0516-7

**Published:** 2019-11-16

**Authors:** Jorge Victorino, Francisco Gómez

**Affiliations:** 1 Departament of System Engineering, Universidad Central, Bogotá, 110311 Colombia; 2 Department of System Engineering, Universidad Nacional, Bogotá, 111311 Colombia; 3 Departamento de matemáticas, Universidad Nacional de Colombia, Bogotá, 111311 Colombia

## Correction to: Plant Methods (2019) 15:11210.1186/s13007-019-0497-6

Unfortunately, the original version of the article [1] contained an error in Figure 7. The names of species *Ulmus minor* and *Acer campestre* were interchanged. The corrected Fig. 7 is given here.

**Fig. 7:**
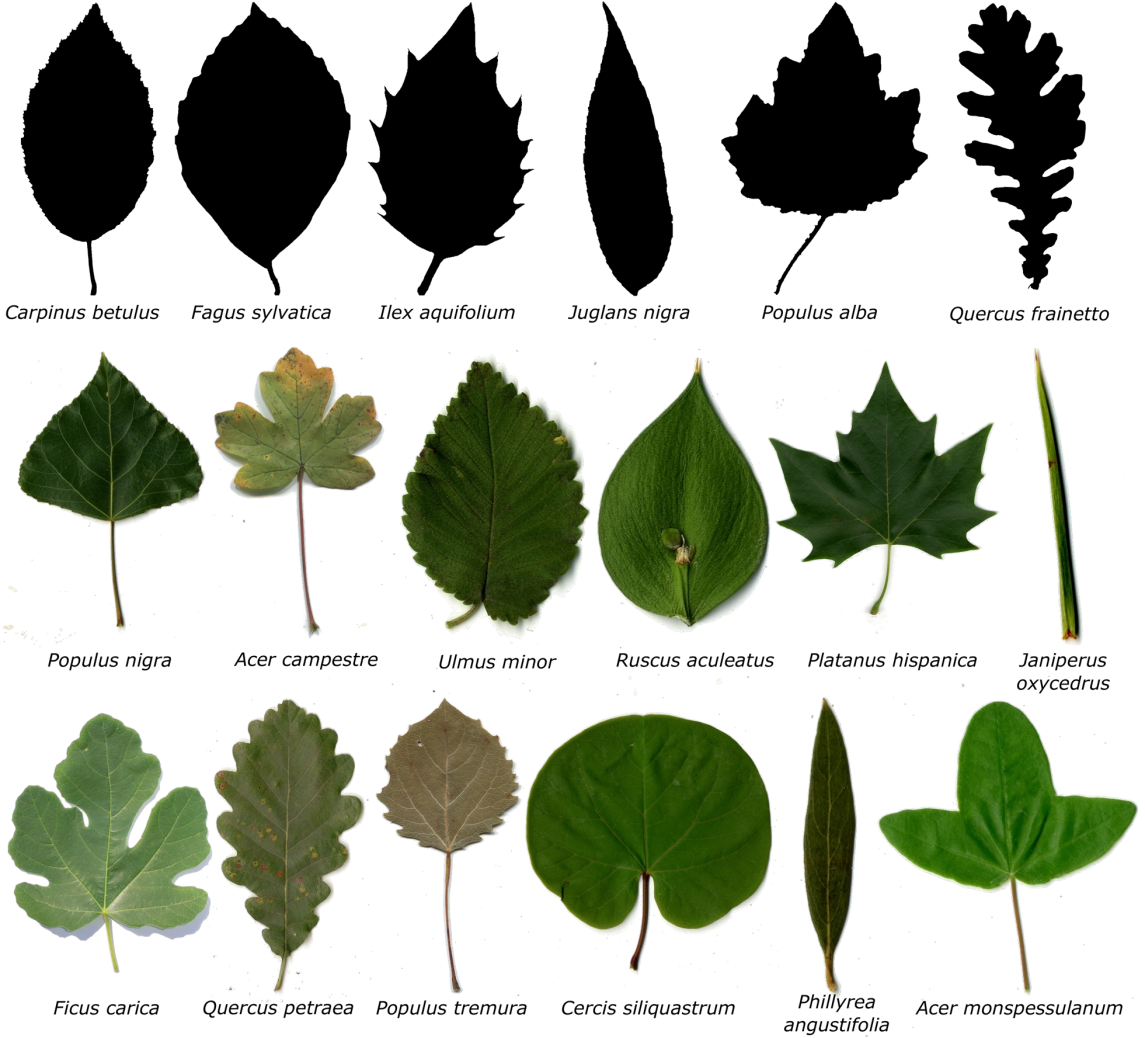
Groups of leaves samples using for testing the method. The image shows the selected species from the TreeMew and ImageClef datasets. The species with the most quantity of samples were selected. The leaves groups were organized in the following way, Top: *Tree leaf database MEW 2010*, middle: *Clef30a* and bottom: *Clef30b*
