# Supplementary material for: Contour analysis for interpretable leaf shape category discovery
Source: Plant Methods. 2019 Oct 7;15:112. doi: 10.1186/s13007-019-0497-6 (PMC6781385; doi:10.1186/s13007-019-0497-6)
Supplement: Supplementary file 1 — Additional file 1. Limits of the category discovery method with multiple samples and species, which reports the results of an experiment aimed to study the limits of the proposed contour analysis method. [file 13007_2019_497_MOESM1_ESM.docx]

**Limits of the category discovery method with multiple samples and species**

This section reports the results of an experiment aimed to study the limits of the proposed contour analysis method in the task of leaf shape category discovery. In particular, the proposal was studied in the task of cluster discovering, when the number of samples and species varied. For these experiments, different datasets were constructed from TreeMew [1] and ImageClef [2] repositories. From these public databases, 25 species were selected. The largest groups corresponded to *Phillyrea angustifolia* species with 168 samples, followed by *Ulmus minor* with 167 samples. The smallest number of specimens per species was 20. The method performance was assessed by the F-score considering as ground truth the sample species.

**The effect of increasing the number of samples per species**

A set of test datasets varying the number of specimens per species was constructed by considering different reference scenarios, namely, with six, four, and two species. In the six species scenario, the number of samples per species varied between 3 and 20 for TreeMew and between 3 and 30 for Clef30a and Clef30b. Table S1 lists the species considered for this evaluation. In the four species scenario, the number of samples per species varied between 3 and 120. In this case, *Buxus sempervirens, Ulmus minor, Ruscus aculeatus, Phillyrea angustifolia* were considered for the evaluation. In the two species scenario, the number of samples per species varied between 3 and 167. For this experiment, *Ulmus minor, Phillyrea angustifolia* were studied.

| **TreeMew** | **Clef30a** | **Clef30b** |
| --- | --- | --- |
| *Carpinus betulus*  *Fagus silvatica*  *Ilex aquifolium*  *Juglans nigra*  *Populus alba*  *Quercus frainetto* | *Populus nigra*  *Acer campestre*  *Ulmus minor*  *Ruscus aculeatus*  *Platanus hispanica*  *Janiperus axycedrus* | *Ficus carica*  *Quercus petraea*  *Populus tremura*  *Cercis siliquastrum*  *Phillyrea angustifolia*  *Acer monspessulanum* |

Table S1. List of species considered to study the effect of varying the number of samples per species.

Figure S1 shows the performance of the method on TreeMew (blue), Clef30a (orange) and Clef30b (green) when the number of samples per species changes in the six species reference scenario.


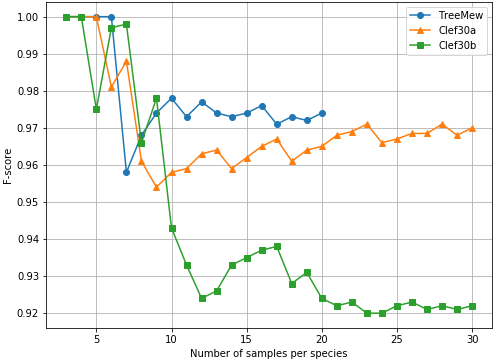


*Figure S1. Performance of the method using TreeMew, Clef30a and Clef30b*

As observed, the method performance remained higher than 0.92 in all the experiments. Remarkably, performances were high, with a small number of samples per species and decreased with more samples per species. However, these performances converged to high F-score values when the number of samples increased, an effect probably associated with performance plateaus.

Figure S2 shows the performance of the method when the number of samples per species varied for the four species (blue) and two species (orange) reference scenarios.


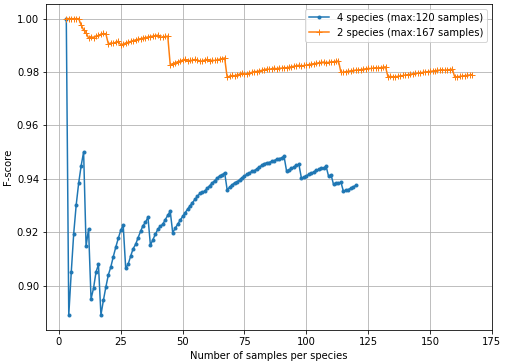


F*igure S2. Performance of the method using four and two species datasets*

As observed, in both scenarios, the method provided a high performance in the shape category detection task. These performances were high, with a small number of samples per species and decreased when the number of specimens per species increased. Nevertheless, these values also converged to high F-score values in both cases, particularly 0.94 and 0.98 for the four and two species scenarios, respectively.

Figure S3 shows the sample distribution on the morphospace for the four species scenario. In particular, the points projected on different PCs (PC1 versus PC2 and PC3 versus PC2) are shown for 3, 5, 10, 20, 40, 60, 90 and 120 samples per species.


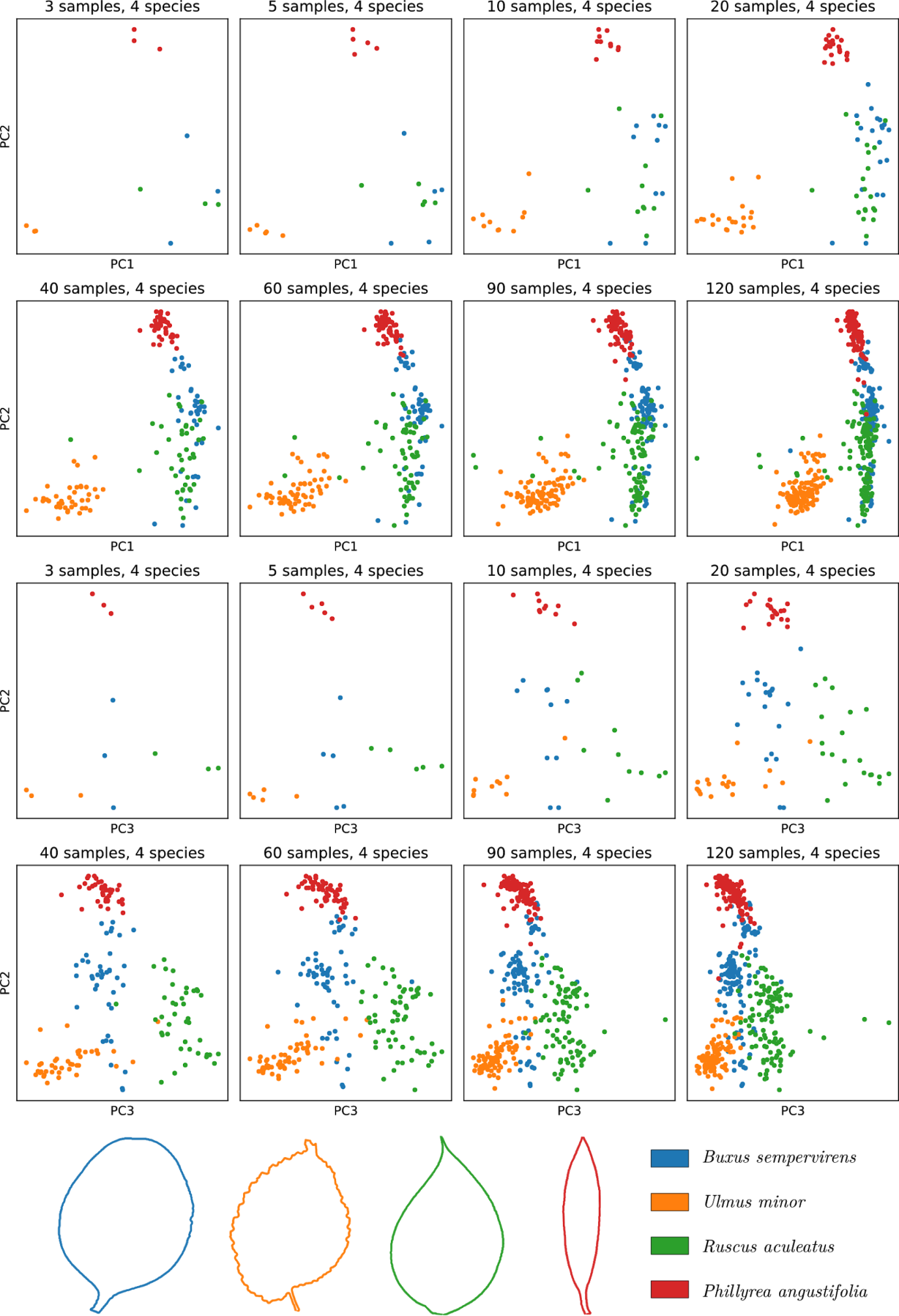


Figure S3. Samples distribution of datasets formed by four species.

As observed, when considering the first two PCs the classes related to *Buxus sempervirens* (blue) and *Ruscus aculeatus* (green) are not well discriminated, but when considering additional components (for instance, PC3), good discrimination of these two classes is reached.

In summary, these experiments show that there are no relevant effects in the method performance when increasing the number of samples per species.

**The effect of increasing the number of shape categories**

A set of test datasets varying the number of species was constructed from a dataset of 25 species. This reference dataset was built by considering species from TreeMew and ImageClef. The test datasets were created, starting with two randomly selected species and increasing one species at a time until reaching 25. For each species, 20 samples were selected. The 25 species included the ones listed in Table S1 together with *Buxus sempervirens, Euphorbia characias, Olea europea, Quercus ilex, Liquidambar styraciflua, Pyracantha coccinea, Quercus robur*. Note that in this experiment, it is assumed that each species represent a particular shape category.

Figure S4 shows the performance in shape category identification when the number of species varied. As observed, performance remains higher than 0.9 for less than nine species and drops for 14 categories. Importantly, this drop in the performance may be linked to a high level of morphological similarity between species. In summary, this experiment shows that there is a dependency between the number of categories to be detected and the method performance.


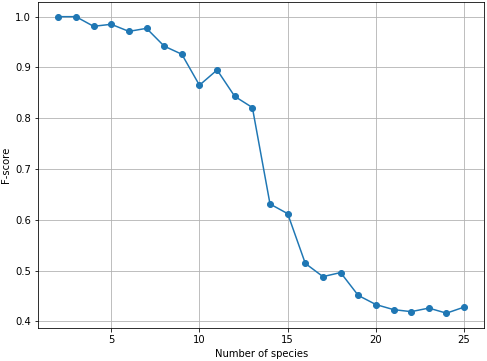


*Figure S4. Performance of the method when the number of clusters varied.*

In conclusion, increasing the number of samples per category has little effect on the performance of the method. On the other hand, increases in the number of categories increase the complexity of the task of category discovery. Although some similarity can be established among samples visually, specific parameter settings should be further investigated.

**References**

1. Suk, T.: Tree Leaf Database, middle european woods MEW. Institute of Information Theory and Automation CAS. Prague, Czech Republic. http://zoi.utia.cas.cz/tree_leaves Accessed 15 July 2019. (2010)
2. Goëau, H., Joly, A., Bonnet, P., Selmi, S., Molino, J.-F., Barthélémy, D., Boujemaa, N.: Lifeclef plant identification task 2014. In: CLEF2014 Working Notes. Working Notes for CLEF 2014 Conference, Sheffield, UK, September 15-18, 2014, pp. 598–615 (2014). CEUR-WS
