# Supplementary material for: Contour analysis for interpretable leaf shape category discovery
Source: Plant Methods. 2019 Oct 7;15:112. doi: 10.1186/s13007-019-0497-6 (PMC6781385; doi:10.1186/s13007-019-0497-6)
Supplement: Supplementary file 2 — Additional file 2. Results reproducibility, which shows how to prepare and run the experiments that reproduce the results of the paper. [file 13007_2019_497_MOESM2_ESM.docx]

**RESULTS REPRODUCIBILITY**

**Construction of datasets**

Three datasets were constructed for experimentation, namely, “TreeMew”, “Clef30a”, and “Clef30b”. These datasets were sampled from two public leaf data repositories: TreeMew (Suk, 2010) and ImageClef (Göeau et al., 2014). The first dataset “TreeMew” is composed of 120 samples from the TreeMew repository. This dataset included samples from six species with at least 20 specimens per species. Table S1 reports the full list of files considered for the database construction. The other two datasets “Clef30a” and “Clef30b” were obtained from the ImageClef repository. Each dataset contained six species with 30 samples per species. The complete lists of files used for the construction of these databases are shown in Table S2 and Table S3 for “Clef30a” and “Clef30b”, respectively.

**Source code**

The file source.rar contains the source code required to reproduce the results of the method for automatic discovery of leaf shape categories reported in the main text. Matlab (version 13) is required to run the experiments. For reproducing results, first the files reported in Tables S1, S2, and S3 should be organized in separate folders. Then unrar the source.rar file in a working folder. The path of the working folder should be added to the Matlab path. Later the file “executeClusterExperim.m” should be modified to associate the locations of the folders containing the leaf database. Finally, the script “executeClusterExperim.m” should run for reproducing the results. The complete lists of scripts correspond to:

- clusterExp.m. A script for executing the experiments of the discovery of leaf shape categories.
- getContorn.m. A script that receives a binary image and extracts the outline of the largest element in the image.
- interparc.m. A script to interpolate the contour to a predetermined number of points.
- adaptiveMeanshift.m. A script for determining the categories given a set of points in a morphospace and a bandwidth.
- performCluster.m. A script that given the clusters and the labels of the points calculates the performance of the method in identifying categories task.
- executeClusterExperim.m. A script to reproduce the reported results.

The ImageClef files require a previous pre-processing stage. For this, use the script “genBinaries.m” to convert file image of ImageClef into a binary image before running the experiments.

*Table S1. Image files selected from the TreeMew repository to construct the TreeMew database. 20 samples per species were selected to construct the test databases.*

| *Carpinus betulus 10.png*  *Carpinus betulus 11.png*  *Carpinus betulus 12.png*  *Carpinus betulus 13.png*  *Carpinus betulus 14.png*  *Carpinus betulus 15.png*  *Carpinus betulus 16.png*  *Carpinus betulus 17.png*  *Carpinus betulus 18.png*  *Carpinus betulus 19.png*  *Carpinus betulus 1.png*  *Carpinus betulus 20.png*  *Carpinus betulus 2.png*  *Carpinus betulus 3.png*  *Carpinus betulus 4.png*  *Carpinus betulus 5.png*  *Carpinus betulus 6.png*  *Carpinus betulus 7.png*  *Carpinus betulus 8.png*  *Carpinus betulus 9.png* | *Fagus sylvatica 10.png*  *Fagus sylvatica 11.png*  *Fagus sylvatica 12.png*  *Fagus sylvatica 13.png*  *Fagus sylvatica 14.png*  *Fagus sylvatica 15.png*  *Fagus sylvatica 16.png*  *Fagus sylvatica 17.png*  *Fagus sylvatica 18.png*  *Fagus sylvatica 19.png*  *Fagus sylvatica 1.png*  *Fagus sylvatica 20.png*  *Fagus sylvatica 2.png*  *Fagus sylvatica 3.png*  *Fagus sylvatica 4.png*  *Fagus sylvatica 5.png*  *Fagus sylvatica 6.png*  *Fagus sylvatica 7.png*  *Fagus sylvatica 8.png*  *Fagus sylvatica 9.png* | *Juglans nigra 10.png*  *Juglans nigra 11.png*  *Juglans nigra 12.png*  *Juglans nigra 13.png*  *Juglans nigra 14.png*  *Juglans nigra 15.png*  *Juglans nigra 17.png*  *Juglans nigra 18.png*  *Juglans nigra 19.png*  *Juglans nigra 1.png*  *Juglans nigra 20.png*  *Juglans nigra 21.png*  *Juglans nigra 2.png*  *Juglans nigra 3.png*  *Juglans nigra 4.png*  *Juglans nigra 5.png*  *Juglans nigra 6.png*  *Juglans nigra 7.png*  *Juglans nigra 8.png*  *Juglans nigra 9.png* |
| --- | --- | --- |
| *Quercus frainetto 10.png*  *Quercus frainetto 11.png*  *Quercus frainetto 12.png*  *Quercus frainetto 13.png*  *Quercus frainetto 14.png*  *Quercus frainetto 15.png*  *Quercus frainetto 16.png*  *Quercus frainetto 17.png*  *Quercus frainetto 18.png*  *Quercus frainetto 19.png*  *Quercus frainetto 1.png*  *Quercus frainetto 20.png*  *Quercus frainetto 2.png*  *Quercus frainetto 3.png*  *Quercus frainetto 4.png*  *Quercus frainetto 5.png*  *Quercus frainetto 6.png*  *Quercus frainetto 7.png*  *Quercus frainetto 8.png*  *Quercus frainetto 9.png* | *Ilex aquifolium 10.png*  *Ilex aquifolium 11.png*  *Ilex aquifolium 12.png*  *Ilex aquifolium 13.png*  *Ilex aquifolium 14.png*  *Ilex aquifolium 15.png*  *Ilex aquifolium 16.png*  *Ilex aquifolium 17.png*  *Ilex aquifolium 18.png*  *Ilex aquifolium 19.png*  *Ilex aquifolium 1.png*  *Ilex aquifolium 20.png*  *Ilex aquifolium 2.png*  *Ilex aquifolium 3.png*  *Ilex aquifolium 4.png*  *Ilex aquifolium 5.png*  *Ilex aquifolium 6.png*  *Ilex aquifolium 7.png*  *Ilex aquifolium 8.png*  *Ilex aquifolium 9.png* | *Populus alba 10.png*  *Populus alba 11.png*  *Populus alba 12.png*  *Populus alba 13.png*  *Populus alba 14.png*  *Populus alba 15.png*  *Populus alba 16.png*  *Populus alba 17.png*  *Populus alba 18.png*  *Populus alba 19.png*  *Populus alba 1.png*  *Populus alba 20.png*  *Populus alba 2.png*  *Populus alba 3.png*  *Populus alba 4.png*  *Populus alba 5.png*  *Populus alba 6.png*  *Populus alba 7.png*  *Populus alba 8.png*  *Populus alba 9.png* |

*Table S2. Image files selected from the ImageClef repository to construct the Clef30a database. 30 samples per species were selected to construct the test databases.*

| *Populus nigra* | *Acer campestre* | *Ulmus minor* | *Platanus hispanica* | *Ruscus aculeatus* | *Janiperus oxycedrus* |
| --- | --- | --- | --- | --- | --- |
| 18228.png  19021.png  19631.png  21182.png  21366.png  21415.png  21510.png  22776.png  22918.png  23037.png  24271.png  24668.png  25070.png  25229.png  25987.png  27229.png  27668.png  27760.png  28171.png  28437.png  30152.png  32170.png  32541.png  32542.png  32819.png  32828.png  32885.png  32977.png  33729.png  35607.png | 18054.png  19614.png  19732.png  20012.png  20212.png  20492.png  21198.png  21277.png  21832.png  22534.png  23736.png  24203.png  24486.png  25844.png  26527.png  28239.png  29359.png  29795.png  29877.png  30443.png  30555.png  31051.png  31896.png  33537.png  33675.png  33874.png  33980.png  34787.png  34808.png  35182.png | 18861.png  18923.png  19568.png  20384.png  21840.png  21861.png  22645.png  22950.png  23092.png  23595.png  23684.png  24388.png  24442.png  24680.png  24712.png  25305.png  25412.png  25450.png  25613.png  25977.png  26091.png  26175.png  27003.png  27080.png  27956.png  28031.png  29479.png  30771.png  31263.png  35798.png | 18223.png  18294.png  19161.png  19203.png  21612.png  23766.png  24048.png  24097.png  25537.png  26359.png  26461.png  27769.png  27934.png  28111.png  28715.png  28793.png  29917.png  30228.png  30752.png  30861.png  30960.png  31020.png  32859.png  33084.png  33367.png  33831.png  33918.png  34280.png  35147.png  35942.png | 18433.png  18508.png  18800.png  19639.png  19655.png  20343.png  20584.png  21234.png  21241.png  21699.png  22481.png  22590.png  23874.png  23990.png  24457.png  24985.png  25646.png  25714.png  25878.png  26080.png  26559.png  27290.png  32579.png  32787.png  33023.png  33986.png  34471.png  34572.png  34679.png  35439.png | 18207.png  18903.png  18955.png  19242.png  19801.png  19818.png  19858.png  20061.png  20495.png  20612.png  20726.png  21016.png  21300.png  21610.png  21757.png  22611.png  22649.png  24407.png  27391.png  27544.png  28818.png  28945.png  29365.png  30128.png  31575.png  34209.png  34230.png  34307.png  34827.png  35920.png |

*Table S3. Image files selected from the ImageClef repository to construct the Clef30b database. 30 samples per species were selected to construct the test databases.*

| *Ficus carica* | *Quercus petraea* | *Populus tremura* | *Cercis siliquastrum* | *Phillyrea angustifolia* | *Acer monspessulanum* |
| --- | --- | --- | --- | --- | --- |
| *18693.png*  *19618.png*  *20303.png*  *20310.png*  *20357.png*  *20473.png*  *20900.png*  *21830.png*  *22078.png*  *22317.png*  *22779.png*  *23815.png*  *24253.png*  *26072.png*  *26379.png*  *27480.png*  *27728.png*  *28525.png*  *30453.png*  *30633.png*  *31339.png*  *32093.png*  *32760.png*  *33347.png*  *34343.png*  *34407.png*  *34594.png*  *34739.png*  *35822.png*  *36146.png* | *18293.png*  *18415.png*  *19362.png*  *19678.png*  *20857.png*  *21787.png*  *22904.png*  *23194.png*  *23485.png*  *23858.png*  *23889.png*  *24251.png*  *24940.png*  *26038.png*  *26780.png*  *27835.png*  *28399.png*  *29560.png*  *29757.png*  *30147.png*  *30835.png*  *30896.png*  *31704.png*  *33750.png*  *34390.png*  *35028.png*  *35099.png*  *35224.png*  *35694.png*  *36246.png* | *18053.png*  *18254.png*  *18773.png*  *20375.png*  *20638.png*  *20819.png*  *21228.png*  *22000.png*  *22421.png*  *22444.png*  *22946.png*  *23193.png*  *23196.png*  *24973.png*  *25397.png*  *25535.png*  *25660.png*  *26185.png*  *27181.png*  *27515.png*  *27703.png*  *29318.png*  *29482.png*  *30380.png*  *30392.png*  *30547.png*  *32041.png*  *32805.png*  *32933.png*  *35748.png* | *18039.png*  *18127.png*  *18621.png*  *21475.png*  *21533.png*  *22088.png*  *23383.png*  *23878.png*  *24178.png*  *24358.png*  *26456.png*  *28576.png*  *28820.png*  *29166.png*  *29733.png*  *29774.png*  *30365.png*  *30402.png*  *31590.png*  *32351.png*  *32408.png*  *32872.png*  *33013.png*  *33032.png*  *33339.png*  *33385.png*  *34344.png*  *34877.png*  *35066.png*  *36052.png* | *18334.png*  *18457.png*  *18491.png*  *18536.png*  *18635.png*  *18657.png*  *18810.png*  *19060.png*  *19075.png*  *22335.png*  *19158.png*  *19174.png*  *19296.png*  *19620.png*  *19869.png*  *20023.png*  *20083.png*  *20802.png*  *20852.png*  *21367.png*  *21532.png*  *21640.png*  *21765.png*  *21874.png*  *21964.png*  *22884.png*  *22061.png*  *22222.png*  *22255.png*  *22389.png* | *18997.png*  *19197.png*  *20078.png*  *20347.png*  *21054.png*  *22860.png*  *23163.png*  *23610.png*  *23725.png*  *24859.png*  *24952.png*  *25317.png*  *25651.png*  *26184.png*  *26205.png*  *27048.png*  *27819.png*  *27992.png*  *28041.png*  *29299.png*  *30998.png*  *31305.png*  *31316.png*  *31805.png*  *32226.png*  *33027.png*  *33662.png*  *34206.png*  *34441.png*  *35597.png* |

*Bibliography*

*Göeau H., Joly, A., Bonnet, P., Selmi, S., Molino, J.-F., Barth ́el ́emy, D., andBoujemaa, N. (2014). Lifeclef plant identification task 2014. InCLEF2014Working Notes. Working Notes for CLEF 2014 Conference, Sheffield, UK,September 15-18, 2014, pages 598–615. CEUR-WS.*

*Suk, T. (2010). Tree leaf database, middle european woods mew. institute of information theory and automation cas. prague, czech republic. http://zoi.utia.cas.cz/tree\_leavesAccessed 15 July 2019*
